# Building and evaluating a social prescribing program for carer-employees: an intervention study

**DOI:** 10.3389/frhs.2026.1918717

**Published:** 2026-08-27

**Authors:** Allison M. Williams, Brooke Chmiel, Kate Mulligan, Sarah Neil-Sztramko, Winny Fung, Srija Biswas

**Affiliations:** 1 School of Earth, Environment and Society, McMaster University, Hamilton, ON, Canada; 2 Dalla Lana School of Public Health, University of Toronto, Toronto, ON, Canada; 3 Department of Health Research Methods, Evidence & Impact, McMaster University, National Collaborating Centre for Methods and Tools, McMaster University, Hamilton, ON, Canada; 4 Canadian Institute for Social Prescribing, Toronto, ON, Canada

**Keywords:** carer-employees, health and wellbeing, intervention research, sequential exploratory mixed methods design, social prescribing

## Abstract

**Background:**

Within a 12-month time window, and in partnership with the Canadian Institute for Social Prescribing (CISP), an extension of the Canadian Red Cross (CRC), we will develop and test a social prescribing (SP) program intervention in Hamilton, Ontario, Canada for Carer-Employees (CEs) - those who balance unpaid care of an adult dependent with paid employment. Given the emergence of SP programs geared towards unpaid carers more broadly, the development of an SP program specific to CEs will provide coordinated access to social and community supports that are unique to the needs of CEs in balancing their dual role.

**Methods:**

OBJECTIVE 1: Informed by a recently completed scoping review of SP programs for CEs, the team will work closely with partner organizations, CISP and the CRC, and a Project Advisory Committee (PAC), including CEs with lived experience, to develop an SP program intervention via a co-design approach drawing on design thinking techniques and collective impact initiative backboned by CISP's SP pathway. The SP program will then be used as an intervention to be tested in the province of Ontario, Canada, via OBJECTIVE 2. OBJECTIVE 2: Using a sequential exploratory mixed-methods design, four Plan-Do-Study-Act (PDSA) cycles (a problem-solving model for improving the SP intervention program) will refine the implementation characteristics of the developed SP program and explore its impacts on CE health. The intervention(s) will operate as a quasi-experiment, where a quasi-experimental pre-post design will be conducted within each PDSA cycle, allowing the SP intervention to be tested and refined with respect to potential adoption by CEs. Impacts on CEs’ health will be determined within each PDSA cycle, specifically on CEs’ mental, psychosocial, and physical health. Quantitative and qualitative data will be generated within each cycle to inform refinements to implementation. A wait-list control group will be randomly assigned to ensure that the observed improvements can be attributed to the intervention.

**Discussion:**

Given the national reach and scope of CISP and the CRC, their commitment to the present initiative provides the project team with the resources necessary to build an effective SP program for CEs in Ontario, while also establishing sustainability through the expansion of this SP program across Canada. The final SP program will be scaled up to the national level.

## Introduction

The majority of carer-employees (CEs), who simultaneously manage paid employment and unpaid care for an adult dependent, are women. Their participation in the workforce provides both social and financial support to their unpaid caregiving role, with the latter being linked with negative mental and physical health outcomes (1). Similarly, fulfilling the challenges of cultural and familial obligations while engaging in paid labour can lead to psychological stress, less time for self-care, and role conflict, as seen in many cultures where women are expected to sacrifice their own needs for the needs of their partner(s), children, aging parents, and in-laws. CEs often encounter role strain in navigating paid work and unpaid care duties, often leading to reduced labour force participation, productivity, quality of care provided, and/or face negative health outcomes (2–5). Published research has revealed a range of negative health outcomes experienced by CEs, including loneliness, social isolation, impacts on emotional well-being, quality of life, general health, self-efficacy, and health care utilization (6–12).

As the latest Statistics Canada 2018 General Social Survey on Caregiving and Care Receiving notes (13), CEs make up 1 in 4 Canadians of employment age (19–70), most of whom work full-time hours, equating to over 5.2 million Canadians. Almost 1 in 3 CEs are 50–59 years old, representing the most skilled and experienced in the workforce. Women more than men reported that caregiving impacts their job security and productivity, with those who provide more than 10 h of unpaid care per week being at higher risk of poor work-life balance and job insecurity. On average, women provide up to 3 h more per week on caregiving than men and often do more of the intense personal care. Women also accounted for almost 60% of all employees who left the labour force due to caregiving and reported missing more days of paid work because of caregiving. Further, 1 in 5 carer-employees make less than $20,000/year (before taxes), making them vulnerable to poverty.

Given the documented role strain and adverse health outcomes CEs face, there is a need for tailored interventions to support the mental and physical health of this growing population. Although the definition of social prescribing (SP) is evolving, programs are known to combat all of these negative outcomes, as noted in a 2022 systematic review (14). SP programs include non-medical prescriptions that strengthen health and well-being, including community and social supports. They can encompass a wide range of activities and supports, such as exercise, mindfulness, meditation workshops, counselling services, and resources associated with assisted meal and transport services. Given the diversity of activities, supports and delivery of SP in Canada (14), the recently completed scoping review (15) was conducted in order to inform the development of a SP program intervention for unpaid carers. SP has been explored for caregivers more broadly, but there appears to be limited research specifically focused on CEs in Canada (16). Given the gap in our understanding of SP options for CEs and unpaid carers more generally, the proposed research will inform the effectiveness of not only the targeted SP activities but also the program characteristics, such as the duration, frequency, and delivery methods. Given CEs' known time and space constraints, a new approach to and model for SP is needed to ensure SP works to improve the health and wellbeing of CEs to stay healthy and sustain their multiple roles, while sustaining optimal quality of life and care (1, 7–12, 14, 16–18). SP programs not only have the potential to assist CEs in effectively navigating the domains of paid work and unpaid care, but also alleviate strain on healthcare systems by reducing healthcare system utilization for health challenges remedied through SP (17). Further, given that SP works best when positioned not only as a navigation tool for individuals but as a mechanism for surfacing systemic gaps in community support and workplace policy (guiding future investment), this work will potentially surface new data about the structural drivers of CE burden (i.e., gendered labour norms, inadequate caregiver-friendly workplace policies, insufficient public services).

The SP program design will be strengthened by an explicit asset-based framing, given that CEs bring significant relational capital, community knowledge, and caregiving expertise/resources that SP programs can amplify rather than simply compensate for. This will be accomplished through ensuring that community assets and strengths that CEs already draw upon are amplified, contributing to program sustainability given that SP programs that build on existing community strengths tend to have better uptake and retention than those framed primarily around risk and remediation. CEs actively engaging in the developed SP intervention program will benefit from being connected to community and social assets/resources to better help them address their needs as they juggle caregiving and paid employment. The SP intervention program will provide CEs with a starting point to access and navigate services available to them without having to do the heavy lifting of searching for these services or opportunities on top of a very busy schedule. The proposed SP intervention will either directly or indirectly address the financial/economic, health, and social vulnerability of CEs. Project Partner Canadian Institute for Social Prescribing (CISP) will be provided the opportunity to expand and scale up the SP intervention to the CE population across Canada, with unique and specific needs around balancing unpaid care and paid employment. Through CISP, this CE-curated SP program will be expanded through other SP initiatives in Canada.

## Methods and analysis

### Ethics

Securing Research Ethics approval (McMaster Research Ethics Board #8113), in accordance with the Tri-Council of Canada's Ethical Conduct for Research Involving Humans – TCPS 2 (2022), the project will start in March of 2026 and conclude in March of 2027. Within this 12-month time window, a Project Advisory Committee (PAC), made of a group of researchers, research assistants, lived experience experts, together with the CISP and Canadian Red Cross (CRC), will meet virtually at least once every 6 weeks throughout the duration of the project. Members of the PAC with lived experience as CEs will receive an honorarium for their expertise and engagement.

### Design

**Figure 1:**
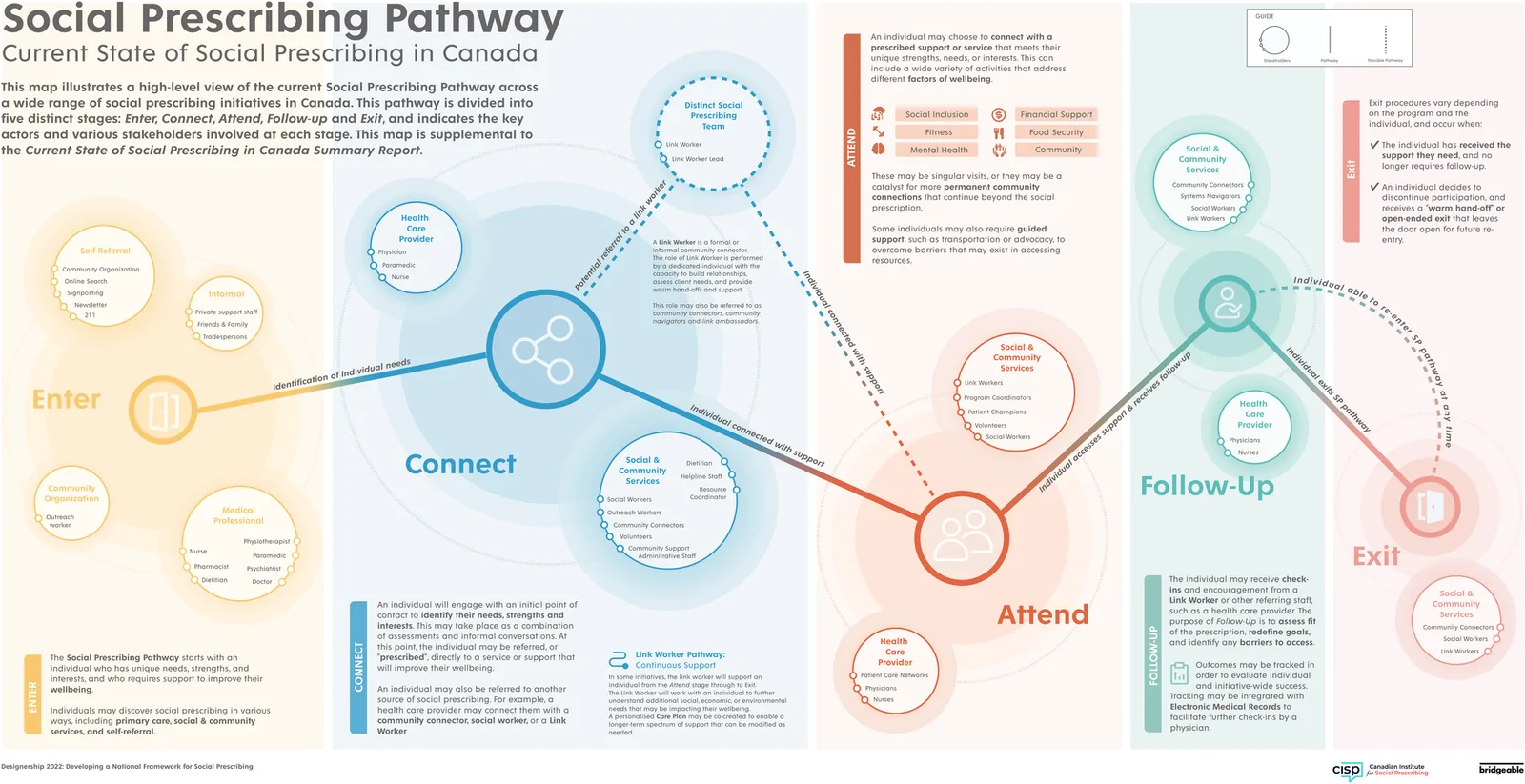
The social prescribing pathway (https://www.socialprescribing.ca/what-is-cisp).

*OBJECTIVE 1: SP Intervention Development (3 months)*: Informed by the SP scoping review (15) the research team will work closely with the PAC members to develop specific design and delivery considerations for a SP program intervention via a design thinking approach, referring directly to the educational resources provided by project partner CISP (https://www.socialprescribing.ca/what-is-cisp). In particular, the foundational knowledge and frameworks, as well as the developed SP Pathway (see Figure 1) will be used to co-build the SP program pilot (19). Building upon previous literature on effective SP programs (14, 16, 17, 20), the intervention will potentially involve connections to existing community-based programs, tools and supports to support physical health (e.g., fitness classes, yoga), mental health (e.g., mindfulness, support groups), social/emotional well-being (e.g., arts, learning new skills), and a variety of other holistic, non-medical services (e.g., financial advice, housing and food insecurity support, employment support, etc.). The developed SP program intervention will be tested as an SP intervention in Objective 2.

The co-creation work will unfold over 4 main phases, consistent with the double diamond of design thinking (21).

### Interventional methods

Phase 1: Discover (0.5 months). The objective of the discovery phase is to understand both what is working well and the pressure points (touch points) in the experiences of CEs, from both the CE and employer perspectives. Touch points are emotionally charged and memorable moments and will be used to characterize the experiences of participants from both the CE and the employer perspective. For example, carer-inclusive employers may have implemented workplace awareness initiatives or introduced carer-friendly workplace policies such as remote options. We will present a range of SP models and be asking what model of SP they will want to adopt. We will conduct key informant interviews (*n* = 7 each from CEs and employers of CEs (not from the PAC) in order to hear from several diverse experiences to identify touchpoints from each perspective. The interviews are expected to last approximately one hour and will primarily be conducted online to accommodate busy schedules, or in-person if preferred. Interviews will be conducted using a semi-structured interview guide that asks informants to share stories about past experiences in their various roles. The research team will use an inductive approach to thematic coding using Atlas.ti qualitative data management software in order to identify touch points, with triangulation across coders. The output from this stage will be a summary of touch points from each perspective. Interview data will be encrypted and only accessible to the research team, with pseudonyms assigned to the interview participants. Participants will be provided an honorarium for their participation.

Phase 2: Prioritize (0.5 months): This phase has two objectives: (1) to identify the most important touch points as priorities for co-creating from each perspective, and to build rapport among participants from each perspective separately in preparation for the co-creation workshop. We will host 2 focus groups (*n* = 8), one per perspective (i.e., one for CEs and another for employers). At each in-person focus group, which will be recorded for transcription, participants will create an experience map of highs and lows in experiences based on the identified touch points and collectively select the most important touch points from their perspectives. The participants of the focus groups will be guided through this process with prompts, following a developed focus group guide. These experience maps by perspective and priority touch points will be summarized by the research team and shared with all participants in advance of the co-creation workshop Phase 3. All physical data collection through the experience maps will be digitized on site to encrypt and share with the research team for analysis; the physical copies will be destroyed one digitized (i.e., double shredded). The focus groups will be held virtually.

Phase 3: Co-create (0.5 months): The objective of this phase is to share values, priorities, and touchpoints from each perspective. We will host an 3-hour, 18-person (including PAC members, health care providers, community-based organizations, SP link workers, SP program managers and teams) co-creation workshop that will facilitate the generation of prototypes that enable participants from both perspectives to work together to ideate solutions/improvements in the form of prototypes (visual representations) of co-created SP approaches to support CEs. Further, the workshop will provide the opportunity to collectively decide which prototypes will proceed to implementation. Here, the role of the link worker(s) will be explored and defined; in addition to determining who fills this role, the referral process and supported pathway to community connection will be operationalized, with processes defined for tracking and measuring the work of the link worker(s). Informed by CISP's Community Asset Mapping Guide for Social Prescribing, a community asset map for Hamilton will be built and shared before and at this workshop. The community asset map will identify which SP-connectable resources actually exist across the city and different neighbourhoods, and where gaps are concentrated, to ensure the program is grounded in what is actually available and accessible to CEs in specific places.

During the first co-creation round, participants from each perspective will work in small groups on a touch point of primary priority to each group. Next, each small group will receive input and feedback from other small groups to augment their prototypes (referred to as a carousel approach). Once both CEs and employer groups are comfortable with co-creation activities, mixed small groups will co-create prototypes on priority issues that are common across both perspectives. The research team will summarize the key themes from the visual prototypes and prepare a report documenting the findings for each case. The workshop will be held in person at McMaster University; all visual prototype materials will be digitized in the workshop and destroyed on site to ensure confidentiality of participant data. Given the complex nature of the workshop, it will not be recorded as audio recording cannot support the various ongoing conversations in the workshop. The workshop will be facilitated by a co-design expert from the McMaster Institute of Research on Aging (MIRA), guided by CISPs theoretical frameworks and conceptual models. Facilitation protocols will include power considerations, to ensure CE voices are not subordinated to employer priorities in mixed groups. For example, facilitators will actively mitigate power asymmetries through sequencing CE-only ideation before entering mixed groups, and/or using anonymous voting on priorities.

Phase 4: Ongoing Co-Design Refinement Groups (1.5 months): Participants at the co-creation event will be invited to participate in ongoing co-design groups to refine the prototypes of an SP program for implementation. This work will determine a variety of characteristics of the SP program, including modality (i.e., in-person, hybrid, etc.), activity range (cognitive, physical, emotive, etc.), intensity (meeting frequency) and duration (length of individual meetings and of the overall program). Given the limited time that CEs have, this process will be sensitive to determine the most acceptable packaging of the SP program, with respect to its efficiency and effectiveness (i.e., flexible scheduling, asynchronous participation, multi-modal communication, and digital navigation supports). To operationally support these limitations that CEs experience in their access to time and resources, a SP program website, or resource hub, will be developed to support the multi-modal access to various tools and resources; this hub will be informed by CISP's Community Asset Mapping Guide for Social Prescribing. The program website will house all necessary materials for accessibility, including meeting recordings, presentations, workshop materials, infographics, lay reports, as well as any other service-related materials. All resources will endeavour to be accessible at a grade 6 level in order to ensure access by various levels of literacy and health literacy. The expected reach of the website spans the local community in Hamilton, through the networks of PAC members, especially project partner CISP, and is available publicly through internet searches. Existing organizational networks will be utilized to promote the website through website postings and newsletters. The Canadian Centre for Caregiving Excellence newsletter has 3,700 subscribers, including stakeholders, government officials, senior executives and caregivers with lived experience, and a following of 1,800 on X. The Ontario Caregiver Organization newsletter maintains a reach of 2,000 subscribers across Ontario and a following of 4,700 on X. In combination with the 140,000 yearly visitors to the nominated principal applicant (NPA's) national project site for Gender, Health and Caregiver-Friendly Workplaces (https://ghw.mcmaster.ca), the expected reach of the website will ultimately span the total capacity of PAC members and the NPA's platform for a total reach of 152,200 across Ontario and Canada, Additionally, the paid advertisements will expand the expected reach to locals across Hamilton beyond the scope of the research team.

*OBJECTIVE 2: SP Intervention Effectiveness Study (7 months)*: Using a sequential exploratory mixed-methods design embedded within four PDSA cycles, Objective 2 will refine the co-designed SP intervention to test the feasibility, acceptability and adoption amongst CEs and explore the effectiveness of the newly co-designed SP intervention to determine impacts on CE health. The intervention(s) will operate like a quasi-experiment, where a quasi-experimental pre-post design will allow the SP intervention to be tested with respect to potential impacts on health, and specifically on CEs mental, psychosocial, and physical health. Qualitative data will be generated in each cycle and used to inform adaptation or refinements to the program delivery model and implementation plan. In order to ensure that the observed improvements can be attributed to the SP intervention, a randomly assigned wait-list control group will be created (22–24). Those on the wait-list will receive access to the SP intervention following the evaluation period.

**Figure 2:**
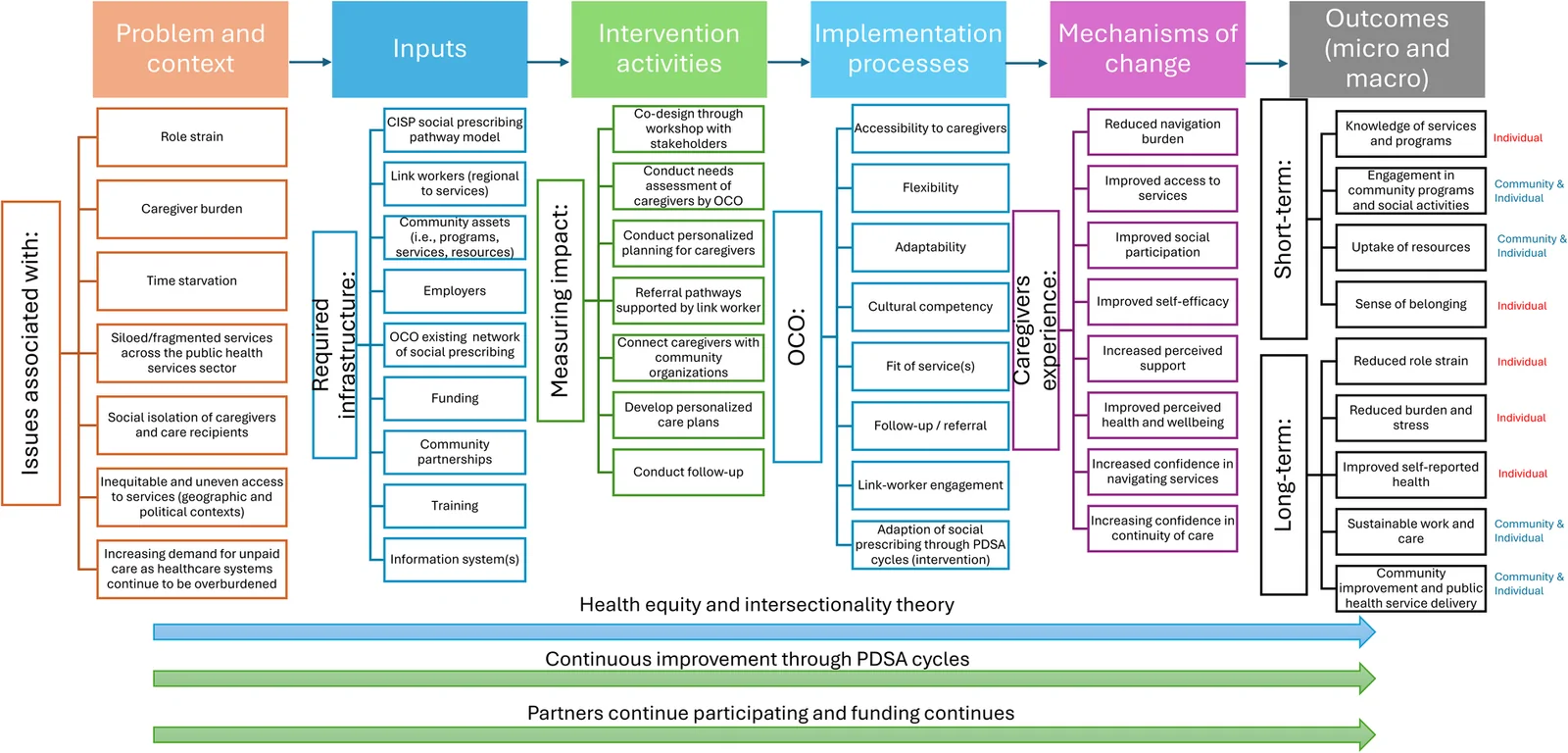
Theory of change program model.

To effectively connect the various conceptual frameworks and methodological approaches guiding this intervention study, a Theory of Change and logic model (ToC) has been created to guide the research team through the implementation of the study within the context of the research objectives. In community based and programming research, ToC and logic models are used to illustrate the long-term goals and outcomes associated with a specific social phenomenon or process (25–30). These models are used to critically assess what is required to facilitate the desired social change and depicts how the complex issue can be addressed through immediate and short-term initiatives and approaches. This often involves collaboration and insight from community partners and members of a research team or initiative, providing clear and actionable steps towards addressing the social change within the context of relevant theoretical frameworks. Figure 2 below illustrates our developed ToC model that situates our SP initiative within the broader context of issues that arise from balancing work and care, outlining the anticipated positive outcomes for CEs in Canada. The model presents the landscape of existing problems around CE strain and lack of effective utilization of community services (Problem and context) that can reduce strain on the healthcare system, leading into the required infrastructure to facilitate better CE health and wellbeing through SP (Inputs). Measuring impact is achieved through the proposed intervention study using the PDSA cycles to continuously adapt the SP program and pathway to better suit the needs of CEs (Intervention activities). The intervention utilized in this study is led by the Ontario Caregiver Organization (OCO) given their existing SP infrastructure including a regional link worker, community pathways and relationships, referral and exit pathways (Implementation processes). These implementation processes facilitate the resources or tools needed to establish change (Mechanisms of change) such as reducing navigation burden for caregivers and facilitating social participation. The anticipated micro and macro outcomes represent the expected impact of the SP initiative around facilitating improvements to CE health and wellbeing and providing proactive responses to care. Applications of equity and intersectionality theory act as a foundation to the developed ToC model by informing the programming and delivery of the intervention to the participant population. In particular, the nuances of caregiver experiences around their own positionality influences their ability to navigate community services and programs, as well as their perceptions of community support and infrastructure. Understanding this informs not only the cultural competency of the degloved SP program but also the variation in experience and need. The figure also provides a logic model illustrating the pathway of change for facilitating better utilization of community services and programs for CEs in Hamilton. The figure connects the existing SP infrastructure of the OCO based on CISPs SP pathway model (see Figure 1) to the intervention study outlined in this protocol. The broader SP pathway supported by the OCO will be leveraged for recruitment of Hamilton based CEs into the intervention study, each PDSA cycle will contribute to refining the selection of services and programs best curated to CEs while also informing our conceptual understanding how to best support CEs in Canada with SP. Recognizing the many CEs in Canada are racialized women, lenses of health equity and intersectionality will be used to shape the pre-post surveys to best inform the SP program.

### Selection/treatment of subjects and data analysis

PDSA cycles originated from the quality improvement literature but have been utilized in the implementation science literature as ways to refine implementation strategies and tailor the mode of delivery to encourage feasibility and adoption across knowledge users. Given the time limitations of the overall project, each of the four PDSA cycle will be 1.5 months in duration. Objective 2 will be the first “Plan”. This will be followed by “Do” (deliver the intervention to a group of CEs over a 4-week period), “Study” (analyze and interpret qualitative and quantitative data in collaboration with the PAC), and “Act” (implement changes in advance of the next program delivery cycle). Defined by a sample size calculation (see below), 40 CE participants will be recruited into each intervention within each PDSA cycle. This will provide a total sample of 160 participants across all four PDSA cycles. A wait-list control group of an additional 40 CE participants will be randomly assigned to ensure that observed improvements can be attributed to the intervention. In addition to collecting socio-demographic data on participants, including sex and gender (via the BSRI), non-invasive health measures will be captured in both the pre-and post-implementation stages, including self-reported health (SF12), self-rated burden (SRB), general self-efficacy (GSE), stress (RSS), mental health (CES-D), and psychosocial health (CRA). The primary outcome is self-reported health. The remaining outcome measures are secondary outcomes. If sex and/or gender are not evenly represented in the participant sample, sex-and/or gender-specific CEs will be targeted. Within the 7-month intervention period, the time between the pre- and post-implementation stages will be reflected by the uptake/application of the intervention(s), both quantitatively [i.e., number of CEs using the intervention(s)] and qualitatively (i.e., intensity of use). Given the experience of other such intervention(s) studies, we hypothesize a minimum 5% increase in self-reported health (SF12). In addition to ensuring that we have a range of sex and gender identities represented when the SP is co-designed, we will be sure to capture participant sex and gender identities in Objective 2. Doing so will allow the determination of how sex and gender impact the experience of the developed SP program. The wait-list control group will receive access to the SP intervention following the evaluation period.

The formula used to calculate the required sample size for the PDSA study design is:

*n* = D[(Z_α+Z_*β*)^2*2*σ^2]/[(*μ*_1-μ_2)]^2

where *n* = sample size required in each time point; μ-1 = the estimated mean of the health measures at the time of the first survey; μ_2 = the expected level of the health measurement at some future date (after intervention), Z_α = the level of statistical significance that the observed change of size would not have occurred by chance; Z_*β* = the statistical power. Based on the published research by Ding, Dardas (31), the mean of the health condition score (SF-12) of CEs was 85. We will use this as the estimated mean of the first interview using standard parameters of: 95% level of significance, and; 80% power and employing a one-tailed test. Suppose an increase of 5% in the health condition score (i.e., SF-12 = 90) is to be measured at the second interview after the SP program is implemented. In this case, μ-1 = 85 and μ-2 = 90; Z_α = 1.96 and Z_*β* = 0.84; *σ*=8, D = 4 to represent the four PSA cycles during implementation.

*n* = 4* [1.96 + 0.840) ^2 *2*80)]/5^2 = 160

Given the size of each of the intervention groups, we do not expect to have any difficulties recruiting 200 (160 CE participants+40 for the wait-list control group), given the research team's extensive network of partner organizations. Social media, together with electronic newsletters and paper posters will be among the recruitment tools used. Even so, we will recruit as many CE participants as possible as a contingency plan. The quantitative data collected will be analyzed by way of descriptive statistics and ordered logit regression. The software SPSS will be used. Summary statistics (including mean tests, cross-tabulations and contingency tables) will be produced to test for significant differences (*p* = 0.05) between pre- and post-test data points within each PDSA cycle. Tests will also be carried out to assess differences between CEs, by various axis of diversity (i.e., sex, geography, etc.). A series of regressions will be carried out to examine the association between self-reported health–SF12 (the dependent variable) and the collected socio-economic variables (the independent variables). It is hypothesized that improvements in pre-post results within each iterative PDSA cycle will be evident as the SP program develops and improves across each PDSA iteration. Missing data will be addressed through imputation calculations for responses missing 30% or less of the data with regards to survey responses (pre-post) (32). Further, it is hypothesized that the social prescribing link workers will become better able to respond to the needs of the CE participants with each iteration of the PDSA cycle. Data will be captured on the number of contacts participants have with the link workers, as well as the contact type (i.e., phone call, electronic message, etc.) and duration of the contact. The results of this analysis will inform the qualitative portion of the research. A sub-sample of participants in each intervention group will be invited to participate in qualitative semi-structured in-depth interviews (*n* = 7) to generate data on the experience of having participated in the intervention(s), capturing the effectiveness of the intervention(s) outside of the quantitative variables examined in the pre-post-test. Following the post-test data collection point, CE participants will be asked about ways in which the intervention(s) have specifically impacted their experience of role strain, caregiver burden and overall health, and aspects of feasibility, mode of delivery, and acceptability. The interviews will be transcribed verbatim, after which they will be thematically coded using Atlas.ti qualitative data management software. Triangulation of both the quantitative and qualitative data will provide confirmability, comprehensiveness and complementarity.

Guided by an intersectionality approach (i.e., sex, gender, racialization, income level, age, social status, educational level, geography) and an equity inclusion perspective, this research will meaningfully address the ways in which social categories, such as sex, gender and employment status (i.e., full-time vs. part-time), intersect to impact CE health. Socio-demographic data will be collected at each stage of the project, allowing for data to be stratified. SP link workers will be involved to assist with referrals, engagement of CE participants with community connection, follow-up/check-ins, identification of barriers and facilitators, and to facilitate data collection on individual-level and community-level outcomes. The work of the SP link workers will be tracked and measured in each PDSA cycle, following the SPHERE model proposed by Kim (33), where SP and health equity are responsively evaluated in order to transform referral systems into adaptive, health equity-driven platforms for structural change (33).

Participants will be provided an honorarium for their participation in both the intervention cycles and the final set of interviews. Encrypted databases will be developed to keep track of participants throughout the various PDSA cycles. It is possible all four PDSA cycles will not be needed, and the final SP program may be developed and finalized after the second or third cycle, at which point the intervention phase of the study would conclude. Only the research team will have access to participant information, and the SP program will only be accessible to those enrolled as a participant in the SP intervention study. Participants will be made aware that they can withdraw from the intervention cycles at any point, and their data will not be used. Once the final SP program is established, it will be made publicly available on the noted resource hub website, moving into the knowledge mobilization activities of the program in collaboration with project partner CISP to scale up the program across Canada.

In addition to individual-level health outcomes, we will also collect pre and post outcome measures related to the Ottawa Charter for Health Promotion and the Quintuple Aim for health care improvement using an equity-stratified analysis, in addition to a healthcare utilization measure, a measure of community capacity building, and a measure of community sense of belonging. This will provide an understanding of how the larger community, health and social care systems have been impacted by the SP program.

### Dissemination

We will build a SP toolkit, which will be available as a free download on the project website. The project website will be developed through the hired services of McMaster University Media Production Services (MPS) through the MacSites platform; the website will act as a resource portal for connecting with in-person services in the community. For the sustainability of the website, the two RAs will be trained on how to update and maintain the website. Recognizing variation in resources across space and place, a generalized SP toolkit will be shared, with key adaptations provided recognizing not a one-size fits all approach.

Upon project completion, the research team lead and trained RAs will continue to maintain the website as a resource portal for CEs in Ontario. Project partner CISP will continue to utilize and promote the resources developed through the project and support the dissemination through their networks, where appropriate. We will market the availability of this toolkit via our social media channels and those of all participating partners/collaborators, through the outlined knowledge mobilization activities within the detailed budget. These include the publishing of two articles to national media channels as well as paid boosts on social media to reach local audiences more quickly in efforts to expand and scale up the SP program.

## Discussion

The primary expected outcome of the program is to directly improve the mental health and wellbeing of CEs as a result of the developed SP program intervention, specific to their ability to better cope with the stresses associated with balancing work and care, as well as better access to resources needed. The goals and objectives of the program surround the provision of a practical and tangible SP program tool that help CEs manage their dual role of providing care to an adult dependent at home or in the community and their paid employment role. Given the rapidly aging population in Canada, coupled with declining fertility rates, the need to support CEs is more economically important than ever (34–38). It is hypothesized that the benefits of the SP intervention will include several health improvements for CEs, such as a decrease in role strain and improvements in CEs' mental, psychosocial and physical health. One key knowledge mobilization strategy is to ensure that the community partner, CISP, will scale up the SP program after the study ends; this will ensure that positive health outcomes will be sustained over time and across the province, and eventually scaled up across the country. In keeping with responsive evaluation of SP and health equity (22), community knowledge specific to unmet needs and service gaps will be channelled into government and NGO policy recommendations, employer engagement, and local advocacy. Taking a community health promotion perspective, we will also include a knowledge mobilization pathway that engages policymakers and employers directly with findings, and that positions CEs themselves as knowledge holders and advocates in the building of carer-inclusive workplaces.
